# Psychosocial distress, perceived need and utilization of psycho- *social* support services in patients in the early phase after the first cancer diagnosis

**DOI:** 10.1007/s00432-025-06107-y

**Published:** 2025-02-06

**Authors:** Hannah Zingler, Diana Steinmann, Jochen Ernst, Ute Goerling, Myriel Hermann, Beate Hornemann, Anja Mehnert-Theuerkauf, Tanja Zimmermann

**Affiliations:** 1 Department of Psychosomatics and Psychotherapy, Hannover Medical School, Carl-Neuberg-Straße 1, 30625 Hannover, Germany; 2 Department of Radiotherapy, Hannover Medical School, Hannover, Germany; 3 Department of Medical Psychology and Medical Sociology, Comprehensive Cancer Center Central Germany (CCCG), University Medical Center Leipzig, Leipzig, Germany; 4 Charité - Universitätsmedizin Berlin, Corporate Member of Freie Universität Berlin, Humboldt-Universität zu Berlin, Berlin Institute of Health, Charité Comprehensive Cancer Center, Berlin, Germany; 5 Comprehensive Cancer Center, University Clinic Centre Dresden, Dresden, Germany

**Keywords:** Psychooncology, Cancer, Psycho-*social* support, Utilization

## Abstract

**Purpose:**

Due to the growing number of new oncological diagnosis and the accompanying psychosocial burden, needs-based psycho-oncological care is important. Adequate planning of psycho-oncological support services is therefore becoming increasingly important. In order to better implement psycho-oncological support services, we investigate psychosocial distress, perceived need and utilization of psycho-oncological support offers in newly diagnosed cancer patients.

**Methods:**

Based on a multicenter prospective study, we assessed the cross-sectional data on psychosocial distress, perceived need and utilization of psycho- *social* support in patients with different tumor entities within 2 months after initial diagnosis. Psychosocial distress was assessed using the Distress Thermometer (DT).

**Results:**

Of 1,003 eligible patients who completed the questionnaire (53.0% men, mean age 60.3 years) 39.7% (*n* = 390) showed above-threshold psychosocial stress (DT: scores ≥ 5) and 21% (*n* = 207) indicated a perceived need for psycho- *social* support. 13.5% (*n* = 136) showed both, psychosocial distress and perceived need for psycho- *social* support. 15.2% (*n* = 150) out of all participating patients used psycho-oncology service, 60.7% (*n* = 597) were willing to accept such an offer. Women were significantly more likely to be psychosocially distressed and to express a need for support. They were also significantly more likely to seek and be willing to accept psycho- *social* support.

**Conclusion:**

Although most patients would accept a psycho- *social* service, regardless of whether there is psychosocial distress or a need is perceived, the actual utilization was relatively low. It can therefore be assumed that barriers, e.g. structural or personal ones, prevent access. These should be investigated in more detail in future studies.

## Introduction

The global number of new cancer cases is known to be high and it is expected to continue to grow over the next years (Bray et al. 2018; Soerjomataram and Bray 2021). Given the multitude of physical symptoms and side effects caused by medical treatments, many patients also report a high level of psychosocial distress, which is why the importance of psycho-oncological care in the treatment of cancer patients is also increasing (Andreu et al. 2022; Brix et al. 2008; Faller et al. 2015; Ostovar et al. 2022). Various studies have shown that about half to two-thirds of cancer patients experience significant psychosocial distress during the course of their disease and about one-third of cancer patients have a mental disorder (Mehnert et al. 2014; Mehnert et al. 2018; Peters et al. 2020). An increased level of distress not only restricts quality of life, but also seems to have a negative influence on the course of the disease (Bortolato et al. 2017; Montazeri 2008; Russ et al. 2012). Psycho-oncological interventions have been proven to reduce psychological symptoms (especially anxiety and depression) as well as improving quality of life with a small to medium effect in meta-analyses and systematic reviews (Faller et al. 2013; Hsieh and Hsiao 2017; Mokhtari-Hessari and Montazeri 2020). Identifying distress after cancer is therefore important to ensure optimal psycho-oncological care.

In Germany, efforts have been made to better address the mental health needs of cancer patients. Within the framework of the National Cancer Plan implemented in 2008 in Germany, the further development of the psycho-oncological care structure was determined. According to this plan, all cancer patients should receive adequate and need-based psycho-oncological care, whereby need-based means not only measurable psychological distress but also the perceived need for psychosocial care (Bundesgesundheitsministerium 2017, 2020). For this, all patients in certified cancer centers should receive a questionnaire on current psychological stress and be offered psycho-oncological support if the threshold value is exceeded. (OnkoZert 2024). In the best practice recommendations on psycho-oncological screening in Comprehensive Cancer Centers (CCC) in Germany by Stengel et al. (2021), the need for psycho-oncological support is defined by the recorded psychological distress of the patients. In particular, this burden is measured by a distress screening as well as the query of psychosocial stress factors. In addition to the psychosocial distress, the perceived need, i.e., the patient’s desire for psycho-oncological support, must also be taken into account for need-based care (Stengel et al. 2021). In addition, the German S3 guidelines for psycho-oncology also recommend not only to use a distress screening but also to ask for perceived need for psycho-oncological care (Deutsche Krebsgesellschaft et al. 2023).

While there are numerous studies on psychosocial distress, there is little research on perceived need in the early survivorship phase. However, previous studies have shown that the presence of psychological symptoms or distress is not necessarily related to the desire (perceived need) for psycho-oncological support. Faller et al. (2016) reported that while one in three cancer patients indicated psychosocial distress, only half of the patients with high psychological distress also showed a perceived need for psychosocial support. Additionally, a quarter of patients without increased psychological distress also desired (perceived need) psycho-oncological support (Faller et al. 2016). In another study, Weis et al. (2018) showed that 30% of the participating cancer patients reported a perceived need. Of these, 61.6% received psychological support (Weis et al. 2018). In further studies, female gender was also identified as an influencing factor on high psychosocial distress, perceived need as well as utilization of psycho-oncological care (Merckaert et al. 2010; Sauer et al. 2019; Singer et al. 2013; Syrowatka et al. 2017; Walker et al. 2020).

In clinical care, however, patients with a high distress value do not necessarily use psycho-oncological services (Baker-Glenn et al. 2011; Clover et al. 2015; Tondorf et al. 2018). Nevertheless, it was not examined so far whether the patients with a high distress also had a perceived need or whether patients with a perceived need accept psycho-oncological support services.

In conclusion, it is known that psychosocial distress measured with a screening instrument and the perceived need for psycho-oncological support are two different constructs that are of great importance for the need-based planning of psycho-oncological support services. However, the findings on perceived need in particular have not yet been sufficiently investigated with regard to utilization of psycho-oncological services. Furthermore, it is still unclear to what extent psychological distress and perceived need overlap. In addition, the recording of psychosocial stress, perceived need and utilization in previous studies is very heterogeneous over the course of the disease and often only relates to individual oncological diseases. Since the mental state at the beginning of a tumor disease is the strongest predictor of mental morbidity during the course of the disease, it is important to be able to intervene as early as possible in order to prevent the manifestation of mental disorders and to maintain quality of life (Boyes et al. 2013; McLouth et al. 2021). Therefore, it is important to record psychological distress and perceived need immediately after diagnosis.

## Study objective

The aim of the present study is to assess psychosocial distress and the perceived need for psycho- *social* support as well as the utilization of psycho-oncological support in an early phase after primary cancer diagnosis. The following research questions will be investigated:

1. How high is the psychosocial distress (suprathreshold score on distress screening), perceived need (desire for support), actual utilization, and willingness to accept psycho-oncology support?
2. Are there gender differences in psychosocial distress, perceived need, actual utilization and willingness to accept psycho-oncology support?
3. Do patients with high psychosocial distress also indicate a perceived need as well as patients with a perceived need indicate high psychosocial distress?
4. Do patients with high psychosocial distress and/or perceived need actually use psycho-oncological support?
5. What is the willingness of patients with high psychosocial distress and/or perceived need to accept a psycho- *social* service?

## Methods

### Study design and procedure

The present data were collected within the framework of the observational study “Longitudinal analysis of psycho-oncological support needs in patients and their relatives, stratified by biopsychosocial influencing factors” (“LUPE”), funded by the German Cancer Aid, in the period from April 2020 to July 2022 (t1). The primary aim of the LUPE-study is to estimate the prevalence of mental disorders, psychosocial distress and perceived psycho-oncological care needs as well as the utilization of psycho-oncological support services in the process from initial diagnosis (maximum 2 months after diagnosis) to follow-up (18 months after diagnosis) as well as the identification of biopsychosocial influencing factors (Mehnert-Theuerkauf et al. 2023). Patients and their relatives were assessed at four measurement time points from diagnosis to follow-up at 6-month intervals. For the present publication, we use the cross-sectional patient data of the first measurement (up to 2 months after diagnosis).

Participating cancer patients were recruited at the three comprehensive cancer centers and study centers (Leipzig, Berlin, Hannover) and at three additional cooperating cancer centers (Dresden, Braunschweig and Göttingen). Inclusion criteria were a confirmed initial diagnosis of malignant solid tumor disease in the last 2 months at the time of participation, a minimum age of 18 years, as well as good German language skills, sufficient cognitive, mental, and physical abilities, and written informed consent. Exclusion criteria were hematologic neoplasms due to the wide variation in tumor type, treatment, side effect, and prognosis. After written informed consent, the medical data were taken from the medical records and patients received a questionnaire with validated self-report instruments. The patients were also interviewed using the Structured Clinical Interview for DSM-5 Disorders (SCID-5-CV) (First et al. 2015). For the present study, only some of the patients’ self-report instruments were evaluated.

### Measurement

Patients received a self-report questionnaire with validated measurement instruments, of which selected ones were analyzed in the present paper, as well as questions on sociodemographic data (Mehnert-Theuerkauf et al. 2023).

Psychosocial distress: To measure the psychosocial distress, the German version of the NCCN Distress Thermometer (DT) (Mehnert et al. 2006) was used. The DT is a screening instrument used worldwide to assess clinically significant levels of distress in cancer patients (Donovan et al. 2014). The DT is an ultra-short screening with one item designed to capture the patient’s overall level of distress over the past seven days (Gessler et al. 2008). Patients rate their distress on a scale from 0 (no distress) to 10 (extreme distress), with a cut-off score of ≥ 5 indicating clinically significant distress. In the present study, patients with a score ≥ 5 were included in the group of patients with high psychosocial distress, whereas those with a score < 5 were included in the group of patients with low psychosocial distress.

In the present study we also used individual items that have already proven successful in previous studies (Faller et al. 2015; Mehnert and Koch 2008) with regard to the following variables:

Perceived need: As in the best practice of Stengel et al. (2021) and in the German S3 guideline (Deutsche Krebsgesellschaft et al. 2023), the perceived need for support is queried in order to record the subjectively desire for support. For this purpose, the item “Do you currently have the desire for psychosocial support?” with the answer options “Yes” and “No” was used.

Utilization: The utilization of psychosocial support services was recorded with the item “Have you used psychosocial support (psychological support/psychotherapy) due to your cancer journey so far?” with the answer options “Yes” and “No”.

Willingness to accept psycho- *social* support: To test the willingness to accept psycho- *social* support offers, the item “Would you accept an offer of psycho-social support?” with the answer options “Yes” and “No” were used.

### Statistical analysis

The analysis was performed with the statistical program SPSS (version 28.0). In addition to descriptive-statistical procedures, chi-square tests and effect sizes were calculated. A Shapiro-Wilk test was calculated to test for normal distribution and the Mann-Whitney U test was used for group comparison. The significance level was set at *p* < .05.

## Results

### Sample

*N* = 3,327 patients who met the inclusion criteria after first medical record check during the study period were contacted, of which *N* = 1,702 fully met the inclusion criteria. *N* = 1,150 (67.6%) agreed to participate in the study, of these, *N* = 1,003 patients actually completed the questionnaire at the first measurement point. *N* = 198 stated it were too burdensome to participate in the study and *n* = 354 were not interested in participation (Goerling et al. 2024).

**Fig. 1:**
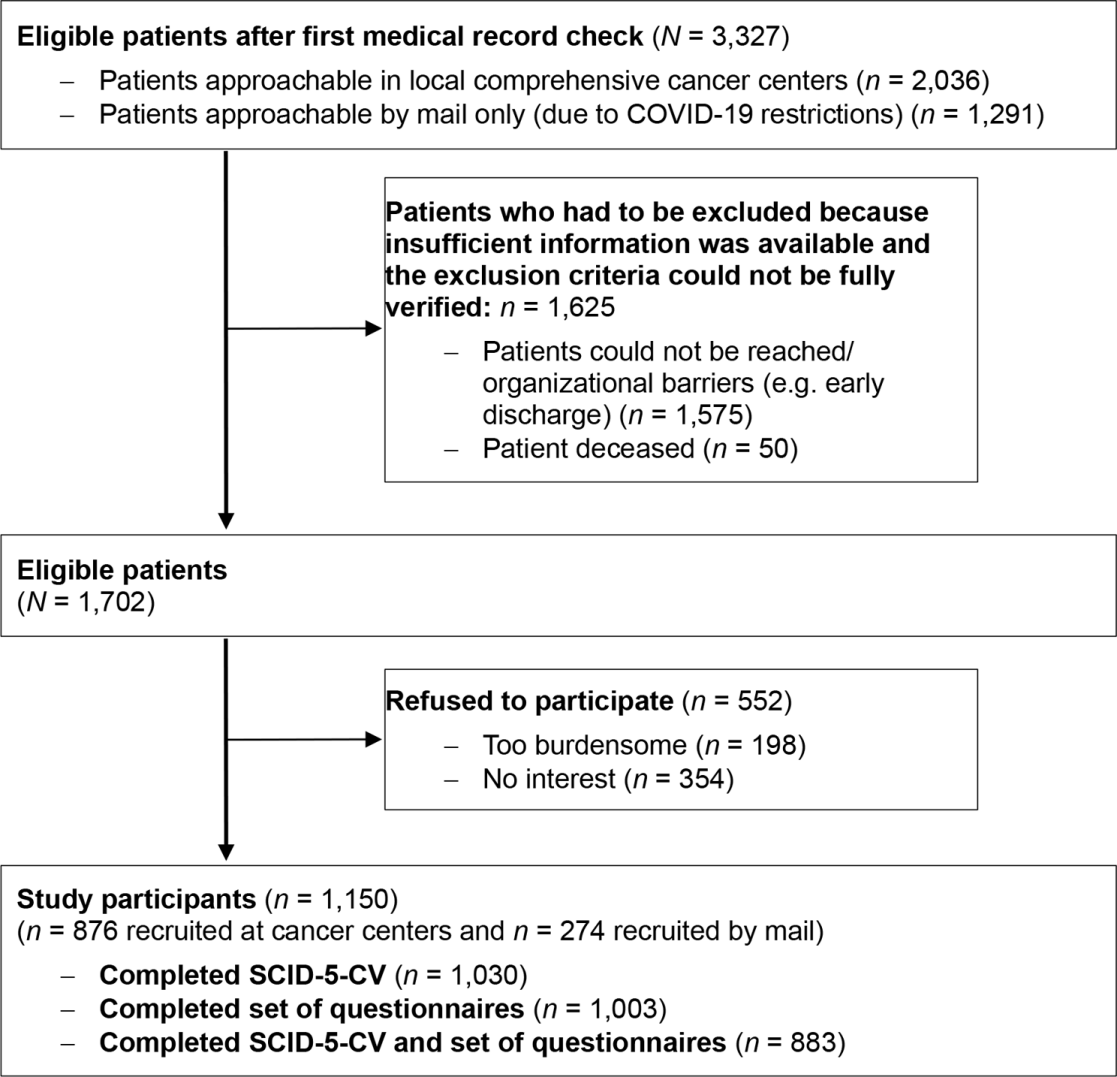
Study flow chart. *Notes*: Study flow chart of the LUPE study see Goerling et al. 2024, COVID = Corona disease, DSM-5 = Diagnostic and Statistical Manual of Mental Disorder, Fifth Edition, SCID-5-CV = Structured Clinical Interview for DSM-5 Disorders – Clinical Version

Participants of the present paper (*N* = 1,003) had a mean age of 60.3 years (SD = 13.1, range: 21–92). 53.9% (*n* = 620) were male. Further characteristics of the participating sample are shown in Table 1.

**Table 1:** Sociodemographic and medical sample characteristics (*N* = 1,003)

		Total (*N* = 1003)		Men (*n* = 532) 53.0%		Women (*n* = 471) 47.0%		*p*		
		n	%	n	%	n	%			
Mean age in years (SD, range)		60.3 (13.1; 21–92)		63.1 (11.9; 21–91)		57.2 (13.7; 22–92)		< 0.001		
Marital status (n, %) Single Married Divorced / separated Widowed		156 597 101 55	17.2 59.5 11.1 6.1	72 336 45 23	15.1 70.6 9,5 4.3	84 261 56 32	19.4 60.3 12.9 7.4	0.017		
Highest school degree (n, %) 9 years 10 years 12 years 13 years No school degree		106 381 73 350 1	11.6 41.8 8.0 38.4 0.1	61 184 31 198 1	12.8 38.7 6.5 41.7 0.2	45 197 42 152 0	10.3 45.2 9.6 34.9 0	0.042		
Employment status (n, %) Employed Unemployed Retired Other		455 21 407 22	50.2 2.3 40.6 2.2	204 11 250 6	43.4 2.1 53.0 1.3	251 10 157 16	57.8 2.3 36.1 3.7	< 0.001		
Tumor site Prostate (C61) Melanoma (C43-C44) Digestive organs (C15-C26) Breast (C50) Female genital organs (C51-C58) Kidney/urinary tract (C64-C68) Head and neck (C00-C14) Lung (C34) Other^a^		188 177 138 131 127 70 61 33 78	18.7 17.7 13.7 13.1 12.7 7.0 6.1 3.3 7.8	188 87 87 3 1 54 39 20 53	35.3 16.4 16.4 0.6 0.2 10.1 7.3 3.8 10.1	- 90 51 128 126 16 22 13 25	- 19.2 10.8 27.2 26.7 3.4 4.7 2.8 5	< 0.001		
UICC stage I II III IV		435 216 175 128	45.6 22.6 18.3 13.4	205 132 99 70	40.5 26.1 19.6 13.8	230 84 76 58	51.3 18.8 17.0 12.9	0.005		

*Notes*: independent-samples *t-test for* significance testing of gender differences in age, Pearson chi-square for testing gender differences in the variables marital status, highest level of schooling, employment status, social class index, and area of residence, ^a^ e.g. C32 - Malignant neoplasm of the larynx, C62 - Malignant neoplasm of the testicle, C76-80 - Malignant neoplasm without indication of localization, UICC stage = tumor classification of the Union internationale contre le cancer

### How high is the psychosocial distress, perceived need, actual utilization, and willingness to accept psycho-social support?

**Fig. 2:**
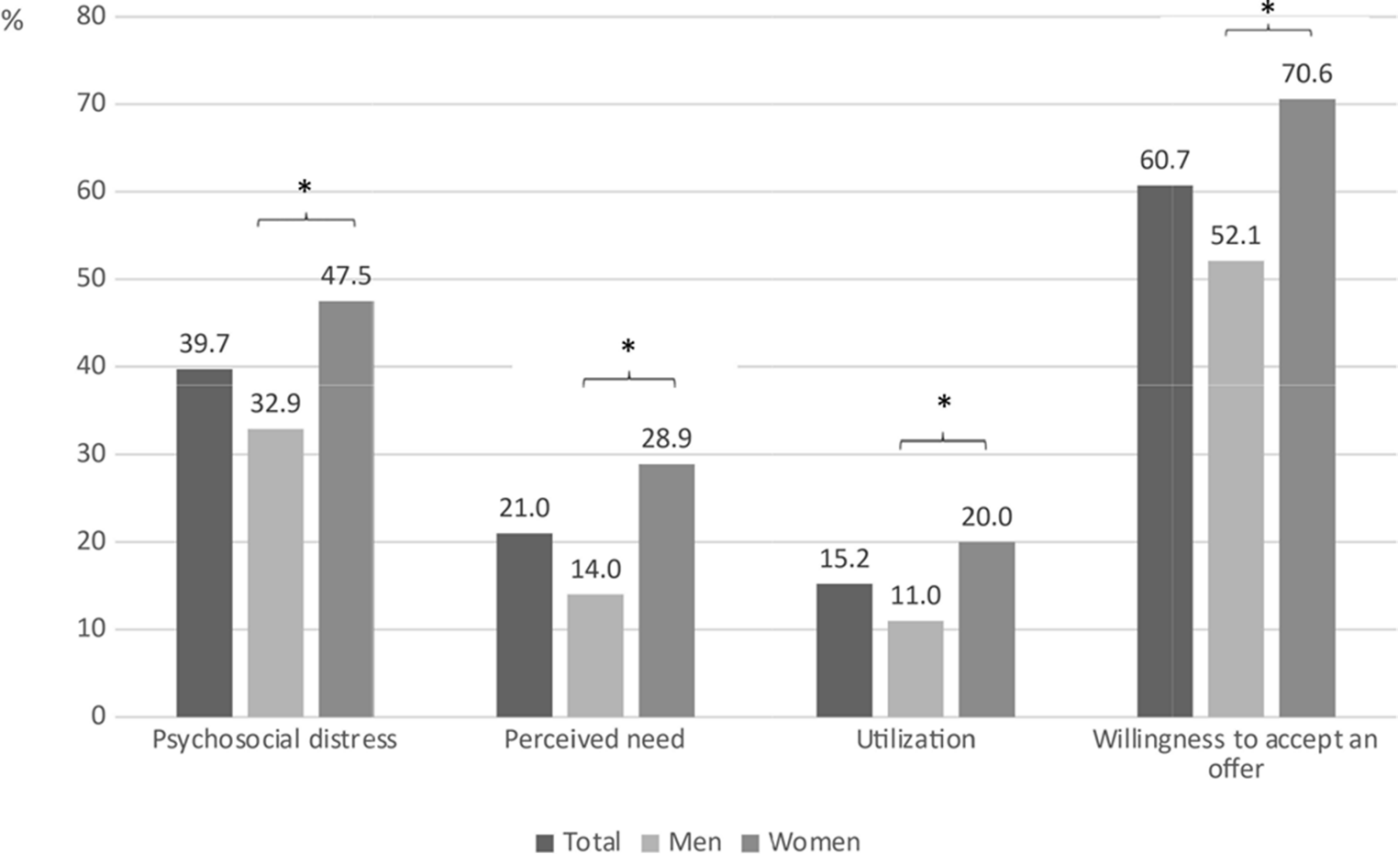
Frequencies of psychosocial distress (DT ≥ 5), perceived need (yes), utilization (yes) and willingness to accept an offer (yes). Data in % (proportion of total sample *N* = 1003), *N* equals., psychosocial distress: distress thermometer with cut-off value ≥ 5, Perceived need = Yes-answer to the question: “Do you currently have a desire for psychosocial support?“, Utilization = Yes-answer to the question: “Have you used psychosocial support (psychological support/psychotherapy) due to your cancer so far?“, Willingness to accept an offer = Yes-answer to the question: “Would you accept an offer of psychosocial support, * = p < .001 (Mann-Whitney U test to compare men and women)

Of all respondents, 39.7% (*n* = 390) indicated high psychosocial distress (DT value ≥ 5) and 21.0% (*n* = 207) indicated a perceived need for psycho- *social* support. Actual utilization of psycho-oncological support was 15.2% (*n* = 150). Willingness to accept psycho- *social* support was affirmed by 60.7% (*n* = 597) (see Figs. 2 and 3).

### Are there gender differences in psychosocial distress, perceived need, actual utilization, and willingness to accept psycho-oncology support?

Women were significantly more likely to show high psychosocial distress (*U* = 102495.0, $\:{n}_{men}$= 523 $\:{n}_{women}$= 459 *p* < .001) and a perceived need (*U* = 103184.5, $\:{n}_{men}$= 527 $\:{n}_{women}$= 460, *p* < .001) than men. The use of psycho-oncological support services (*U* = 109940.0, $\:{n}_{men}$= 525 $\:{n}_{women}$= 460 *p* < .001) and willingness to accept a psycho-oncology service were also significantly higher among women than men (*U* = 98023.5, $\:{n}_{men}$= 524, $\:{n}_{women}$459, *p* < .001; see Fig. 1).

### Do patients with high psychosocial distress also indicate a perceived need as well as patients with a perceived need indicate high psychosocial distress?

Overall, 136 (13.5%) respondents expressed both high psychosocial distress and a perceived need. 248 patients (32.5%) indicated only high psychosocial distress and 68 (11.7%) respondents indicated only a perceived need (see Fig. 3).

### Common intersection of psychosocial distress and perceived need

**Fig. 3:**
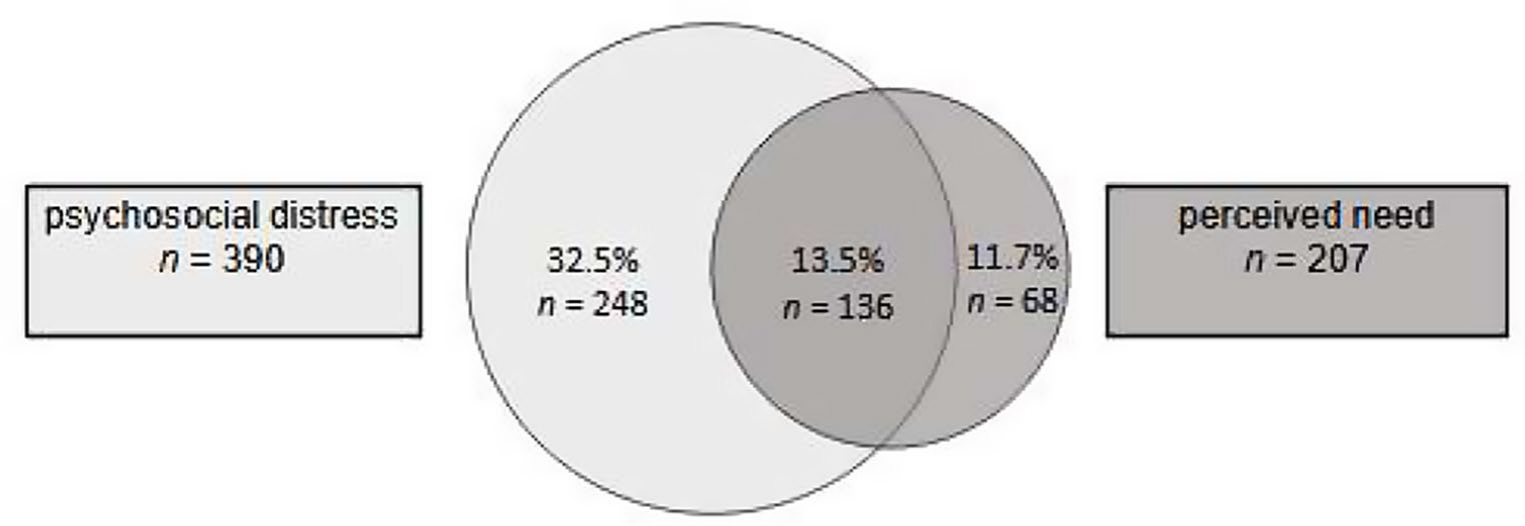
Common intersection of psychosocial distress and perceived need. Psychosocial distress: mean score of Distress Thermometer with cut-off value ≥ 5, perceived need: Yes-answer to the question: “Do you currently have a desire for psychosocial support?”

### Do patients with high psychosocial distress and/or perceived need actually use psycho-oncological support?

Out of the patients with high psychosocial distress 22.6% (*n* = 86, $\:{\chi\:}_{total}^{2}$ (1, $\:{N}_{total}$ = 964) *=* 24.411, *p* < .001) actually used such a psycho-oncological support after diagnosis. Among patients with a perceived need, 36.8% (*n* = 75$\:,\:{\chi\:}_{total}^{2}$ (1, $\:{N}_{total}$ = 973) *=* 91.587, *p* < .001) took advantage of psycho-oncological support services (see Table 2).

**Table 2:** Use of psycho-oncological support in the case of psychosocial distress and/or perceived need

Psychosocial Distress	Utilization				
	Yes (*n* = 149)	No (*n* = 815)	Perceived need	Yes (*n* = 149)	No (*n* = 824)
	*n* (%)	*n* (%)		*n* (%)	*n* (%)
Total					
Yes (*n* = 381)	86 (22.6)	295 (77.4)	Yes (*n* = 204)	75 (36.8)	129 (63.2)
No (*n* = 583)	63 (10.8)	520 (89.2)	No (*n* = 769)	74 (9.6)	695 (90.4)
Male					
Yes (*n* = 170)	27 (15.9)	143 (84.1)	Yes (*n* = 73)	20 (27.4)	53 (72.6)
No (*n* = 346)	31 (9.0)	315 (91.0)	No (*n* = 448)	38 (8.5)	410 (91.5)
Female					
Yes (*n* = 211)	59 (28.0)	152 (72.0)	Yes (*n* = 131)	55 (42.0)	76 (58.0)
No (*n* = 235)	32 (13.5)	205 (86.5)	No (*n* = 321)	36 (11.2)	285 (88.8)

Note: Chi-square test, Utilization: Yes-answer to the question: “Have you used psychosocial support (psychological support/psychotherapy) due to your cancer so far?“, Psychosocial distress (value of Distress Thermometer ≥ 5): $$\:{N}_{total}$$*=* 964,$$\:\:{N}_{men}=516,$$$$\:{N}_{women}$$*=* 448, *n =* actual number, *% =* percentage, *df =* 1, *Pearson Chi-sq.*$$\:{\chi\:}_{total}^{2}$$ 24.411, $$\:{p}_{total}$$ < 0.001, *Pearson chi-sq.*$$\:{\chi\:}_{men}^{2}\:$$*=* 5.476, $$\:{p}_{men\:}$$= 0.019, *Pearson Chi-sq.*$$\:{\chi\:}_{women}^{2}\:$$= 14.419, $$\:{p}_{women}$$*=* 0.001; Perceived need (“Do you currently have a desire for psychosocial support?“): $$\:{N}_{total}$$*=* 973,$$\:\:{N}_{men}=521,$$$$\:{N}_{women}$$*=* 452, *n =* actual number, *% =* percentage, *df =* 1, *Pearson chi-sq.*$$\:\:{\chi\:}_{total}^{2}$$*=* 91.587, $$\:{p}_{total}$$*<* 0.001, *Pearson chi-sq.*$$\:{\chi\:}_{men}^{2}\:$$*=* 22.701, $$\:{p}_{men\:}$$< 0.001, *Pearson chi-sq.*$$\:{\chi\:}_{women}^{2}\:$$*=* 54.779, $$\:{p}_{women}$$*< .*001

### What is the willingness of patients with psychosocial distress and/or perceived need to accept a psycho- social service?

Out of the patients with high psychosocial distress 73.6% (*n* = 282, $\:{\chi\:}_{total}^{2}$ (1, $\:{N}_{total}$ = 963) *=* 41.604, *p* < .001) would be willing to accept an offer of psychosocial support. Among patients with a perceived need for psycho- *social* support 98.5% (*n* = 202, $\:{\chi\:}_{total}^{2}$ (1, $\:{N}_{total}$ = 977) *=* 157.126, *p* < .001) would be willing to accept an offer of psychosocial support (see Table 3). In addition, half of all patients without psychosocial distress (52.9%, *n* = 307$\:{\chi\:}_{total}^{2}$ (1, $\:{N}_{total}$ = 963) *=* 41.604, *p* < .001) and 50.4% (*n* = 389, $\:{\chi\:}_{total}^{2}$ (1, $\:{N}_{total}$ = 977) *=* 157.126, *p* < .001) without a perceived need are willing to accept an offer. Irrespective of psychosocial distress and perceived need, the majority of tumor patients would willing to accept an offer of psychosocial support if they were offered one.

**Table 3:** Willingness among patients with psychosocial distress and/or perceived need to accept a psycho- social service

Psychosocial distress	Willingness to accept an offer				
	Yes (*n* = 589)	No (*n* = 374)	Perceived need	Yes (*n* = 591)	No (*n* = 386)
	*n* (%)	*n* (%)		*n* (%)	*n* (%)
Total					
Yes (*n* = 383)	282 (73.6)	101 (26.4)	Yes (*n* = 205)	202 (98.5)	3 (1.5)
No (*n* = 580)	307 (52.9)	273 (47.1)	No (*n* = 772)	389 (50.4)	383 (49.6)
Male					
Yes (*n* = 169)	108 (63.9)	61 (36.1)	Yes (*n* = 73)	72 (98.6)	1 (1.4)
No (*n* = 344)	162 (46.8)	184 (53.2)	No (*n* = 449)	199 (44.3)	250 (55.7)
Female					
Yes (*n* = 214)	174 (81.3)	40 (18.7)	Yes (*n* = 132)	130 (98.5)	2 (1.5)
No (*n* = 234)	145 (62.0)	89 (38.0)	No (*n* = 323)	190 (58.8)	133 (41.2)

*Note: Chi-square test*, acceptance offer: Yes-answer to the question: “Would you accept an offer of psychosocial support?“, psychosocial distress (value of Distress Thermometer ≥ 5): $$\:{N}_{total}$$*=* 963,$$\:\:{N}_{men}=515,$$$$\:{N}_{women}$$*=* 448, *n =* actual number, *% =* percentage, *df =* 1, *Pearson Chi-sq.*$$\:{\chi\:}_{total}^{2}$$*=* 41.604, $$\:{p}_{total}$$ < 0.001, *Pearson chi-sq.*$$\:{\chi\:}_{men}^{2}\:$$*=* 13.288, $$\:{p}_{men\:}$$*<* 0.001, *Pearson chi-square*$$\:{\chi\:}_{women}^{2}\:$$*=* 20.397, $$\:{p}_{women}$$*<* 0.001$$\:,$$ perceived need (“Do you currently have a desire for psychosocial support?“): $$\:{N}_{total}$$*=* 977,$$\:\:{N}_{men}=522,$$$$\:{N}_{women}$$455, *n =* actual number, *% =* percentage, *df =* 1, *Pearson chi-squared*$$\:{\chi\:}_{total}^{2}$$*=* 157.126, $$\:{p}_{total}$$ < 0.001, *Pearson chi-square*$$\:{\chi\:}_{men}^{2}\:$$*=* 74.190, $$\:{p}_{men\:}$$*<* 0.001, *Pearson chi-square*$$\:{\chi\:}_{women}^{2}\:$$*=* 70.638, $$\:{p}_{women}$$*<* .*001*

## Discussion

In the present study, 1,003 patients, within 2 months of initial diagnosis of cancer, were assessed for their psychosocial distress, perceived need for psycho- *social* support, actual utilization, and willingness to accept psycho- *social* services.

The results show that the psychosocial stress in the present study, at 39.7%, is comparable to the DT values of other studies (Carlson et al. 2019). Nevertheless, studies with significantly higher DT values can also be found in the literature (e.g., Mehnert et al. (2018) 52% or Peters et al. (2020) 65.9% of cancer patients had a DT score ≥ 5). However, the values are difficult to compare with the results of the present study, as the measurement points and recruitment locations vary greatly. “In addition to the different times of the survey during the course of the disease, there were major differences in the sample, particularly with regard to the UICC stages. While more patients with UICC stage I were examined in the present study (45.9% vs. Mehnert et al. 2018 14.9% vs. Peters et al. 2020 30.0%), more patients with higher UICC stage (IV) were examined in the comparative studies (13.4% vs. Mehnert et al., 2018 21.5% vs. Peters et al. 2020 21.5%). The distribution of the types of cancer among the respondents also differed greatly in the available samples. Further studies on the influence of stage and type of cancer on the psychosocial distress, perceived need and utilization are therefore important.”

The perceived need for psycho- *social* support was lower (21%) compared to other studies (e.g., Faller et al. (2016): 32.1% of patients showed a perceived need). A possible explanation could be that in the present study, participants were questioned close to the time of diagnosis, so that the patients may not yet be processing the diagnosis at the time of the interview, but rather are in the shock or reaction phase. Cullberg et al. (2008) define the four phases of shock, reaction, processing, and reorientation for a traumatic crisis, of which cancer is one. In the reaction phase, in which the respondents were at the first measurement point, in particular medical factual information are necessary to re-establish some order or control, which is often disturbed by a cancer diagnosis. Distraction and defense mechanisms are often used to deal with the stress in this phase of illness (Cullberg et al. 2008). Complementary to this, patients are often in autopilot mode during the acute treatment of cancer, where the treatment is gone through without really realizing it (Dreismann and Zimmermann 2021). Considering these factors, the comparatively low number of individuals with high psychosocial distress and/or a perceived need for psycho- *social* support compared to other studies can be explained. On the other hand, a study by Vogt et al. showed that psychosocial distress decreases over the course of the disease. However, it is also unclear here how much time elapses between diagnosis and completion of the questionnaire (Vogt et al. 2021). In order to examine the progression of distress over the course of the disease, the later measurement times of the LUPE study should be used in further studies.In addition, in the above-mentioned comparable studies, there is no fixed time criterion between diagnosis and interview, so that no conclusions can yet be drawn at which point of coping with the disease the psychosocial distress and the perceived need for psycho- *social* support is greatest. Nevertheless, the results should not obscure the fact that almost 40% of cancer patients in acute care show high distress and should therefore also be offered psycho-oncological care.

The analysis regarding the question whether patients with psychosocial distress and/or a perceived need use psycho-oncological support, show that only 22.6% of the patients with a high distress screening and 36.8% of the patients with a perceived need for support also use psycho-oncological support. The question thus arises as to the causes of the low utilization despite the psychosocial distress and perceived need. Reasons can be structural barriers as well as personal reasons. In addition to fear of stigmatization, concerns about destabilization and excessive confrontation with the disease are named as personal barriers (Farkas and Andritsch 2018). Possibly also due to physical weakness caused by the disease, the focus on medical treatment is classified as more superficial than psycho-oncological co-treatment (Fügemann et al. 2022). However, since 98.5% of patients with a perceived need for psycho-oncological support and 73.6% with psychosocial distress stated that they would be willing to accept a psycho-oncological offer, it can be assumed that structural barriers are responsible for the low utilization.

These structural barriers include, for example, lack of information and education on psycho-oncological support services (Bayer et al. 2020; Pichler et al. 2022). In a study addressing this issue, only 38% of respondents reported feeling well-informed about psycho- *social* support options, whereas regarding the medical facets of their disease, 72–88% felt well-informed (Faller et al. 2016). Pichler et al. (2022) showed that in addition to the lack of knowledge about appropriate services, the mode of action of psycho- *social* support was often unknown. In addition, further barriers arise in the recording and documentation of the corresponding need for care (Pichler et al. 2022). In Germany, the S3 guideline on psycho-oncology recommends that all oncological patients receive a screening questionnaire to identify psychological distress and the perceived need for psycho-*social* support and if there are any abnormalities in this screening, patients should be offered psycho-oncological support services. (Leitlinienprogramm Onkologie (Deutsche Krebsgesellschaft 2023). In addition, the certification criteria for oncology centers or organ cancer centers in Germany require screening for psychosocial stress. In a recently published best practice, recommendations for the implementation and performance of such a screening are given (Stengel et al. 2021). Screenings have long been shown to be helpful in communicating about psychological distress between patients and practitioners and therefore to improve information and access to psychooncological support services (Carlson et al. 2012; Esser et al. 2022). Nevertheless, practice shows, that such screenings are often not applied to every patient. Dreismann et al. (2021a, b) found in a study with oncology nurses that although the nurses surveyed considered the screening tool to be very important, the majority of respondents relied on their own clinical impressions when assessing distress and did not use the screening tool. In addition to structural barriers to the implementation of the screening in daily work life, personal barriers were also found, such as a lack of psycho-oncological expertise (Dreismann et al. 2021a). The lack of knowledge and use of screening results in patients with psychosocial distress or perceived need for psycho-oncology support not being adequately identified and educated about appropriate options. It could be helpful to better educate oncology professionals regarding psycho-oncology screening and appropriate support services in order to promote the use of screening and counteract the lack of education of oncological patients. In a qualitative study with expert interviews of nurses, it was shown that there is a need for training regarding psycho-oncological screening among nurses (Dreismann et al. 2021b). Since such studies not only improve information about psycho-oncological support services, but also increase the utilization of such services by patients under stress, the training of specialist staff should be promoted more (Esser et al. 2022).

In addition, the present study not only shows that there are obviously structural barriers which must be further reduced, but that also other factors influencing utilization must also be identified. Although only 18% (*n* = 207) indicated a perceived need of psycho- *social* support and 33.9% (*n* = 150) expressed psychosocial distress, 51.9% of all respondents would accept a corresponding psycho- *social* offer. It is impressive that 50.4% (*n* = 389) of those without a perceived need and 52.9% (*n* = 307) without psychosocial distress would nevertheless accept such an offer. From this it can be concluded that not only the psychosocial distress or perceived need, but also other influencing factors have an effect on the utilization. Furthermore to prevent psychological stress during the course of the disease, it would also make sense to offer patients preventive measures such as psychoeducation. A study with breast cancer patients showed that psychoeducation alone was able to reduce the patients’ psychological distress (Mustikaningsih et al. 2024).

As well as the psychosocial distress and the perceived need also the actual utilization and willingness to accept a psycho-oncology service were significantly higher among women than among men in the present sample. Thus, gender appears to be an important influencing factor on both need and utilization, which is consistent with the results from other studies (Bayer et al. 2020; Peters et al. 2020; Zeissig et al. 2015a, b). Mehnert et al. (2018) reported that in a study of 3724 cancer patients, women in particular showed a higher score on the distress thermometer. Furthermore, men in particular were significantly less likely to use cancer counseling services, of which middle-aged men in particular had the lowest number of psycho-oncological contacts (Bayer et al. 2020; Thode et al. 2005). In a study of breast, colorectal, and prostate cancer survivors, women were twice as likely as men to seek cancer counseling services, and among colorectal cancer participants, women were also more likely to use the psycho-oncology consultation service (11% vs. 7%, *p* =.03) (Zeissig et al. 2015a, b). Zeissig et al. (2015a, b) further demonstrated that women were generally more likely to receive inpatient psycho-oncology care. It can therefore be assumed that gender has an influence on the psychosocial distress, the perceived need and the utilization. As gender differences are consistently found in the literature, gender should also be taken into account in the treatment provided by psychosocial services.

Other factors influencing psychological distress have also already been investigated in some studies. In cancer patients, in addition to female gender, poor physical health, advanced disease status or perceived functional limitations, as well as little social support and low socioeconomic status, have a negative impact on psychological distress in affected individuals (Sauer et al. 2019; Singer et al. 2013; Syrowatka et al. 2017; Walker et al. 2020). However, the extent to which these factors also influence the perceived need and utilization has not been studied in detail. Therefore, it is necessary to identify possible influencing factors in order to be able to establish a need-based psycho-oncology care. It would also be desirable to investigate whether the influencing factors vary in the course of the disease.

Limitations of this research are that the results of the study only refer to individual questions of the patients’ self-assessment and a falsification by e.g. social desirability cannot be excluded. In further studies, therefore, the interview data should also be reviewed and taken into account. A further limitation of the study is the lack of data on non-responders. Even though it is known that the non-responder were older (mean age 65.9 versus 60.4 years, *P* < .001), had poorer performance status (73.6 versus 80.0, *P* < .001) and were more likely to have a low social economic status (SES) (low SES: 27.4% versus 16.6%, high SES: 26.8% versus 38.9%, *P* < .001) compared to study participants (Goerling et al. 2024), there are no information about the psychological distress, perceived need and utilization of these patients, so that a selection bias cannot be ruled out.

“Furthermore limitation results from the wording of the questions regarding perceived need, utilization and willingness to accept an offer. While utilization was explicitly asked about the use of psychological support or psychotherapy, the other two items asked about psychosocial support. This includes besides psycho-oncological support also help from social workers, self-help groups, pastoral support and others. In the items perceived need and willingness to accept, however, these were not further differentiated, so this may have distorted the results. Besides, the term psycho-oncology is not a protected term and is used synonymously with psychological support in the study. Nevertheless even if it is to be expected that utilization will be higher if other psychosocial services are included, it is to be expected that the difference between utilization and willingness to accept a service will remain large. It should also be noted that it is often not possible for the general population to differentiate between the various psychosocial services. The evaluation of the item “willingness to accept psycho-oncological support " (“Would you accept an offer of psychosocial support?“) also showed that further specification would be useful for this question. For example, it could be asked under which conditions (e.g. time, occurrence of stress, etc.) the patients would accept an offer.

A further limitation arises from the use of the distress thermometer. Although the distress thermometer is used internationally, Mehnert et al. (2006) showed only a moderate specificity of 41–47%. It can therefore be assumed that patients without clinical distress are also identified as distressed by the Distress Thermometer. The analysis of the SCID data of the present population showed that 20.9% of the patients had a mental disorder (Goerling et al. 2024). Further evaluations should investigate the extent to which overthreshold distress is associated with a mental disorder. In addition, only limited information can be obtained from the present results due to the use of univariate analyses. In particular, information on the influence of socioeconomic factors on the variables investigated or the influence of diagnoses (made through the SCID interview) is lost. Additional influencing factors should therefore be analyzed in further calculations. Nevertheless Goerling et al. showed that socioeconomic status had no influence on mental disorders in cancer patients (Goerling et al. 2024).

Another limitation results from the data collection in the various centers. Due to the coronavirus pandemic, there were numerous restrictions on personal contact. However, these varied in severity from center to center, so that in some centers it was only possible to recruit by letter at times, while in other centers only some stations were affected by closures. This also has an impact on the generalization of the data, as the population reached does not correspond to the actual population to be reached.

In conclusion, the difference between the utilization of and the willingness to accept an offer can be emphasized. Although in all certified cancer centers every cancer patient should be tested for psychosocial distress and, in the case of high psychosocial distress, services should be utilized, only 22.6% (36.8% with perceived need) of patients with high psychosocial distress utilized psychosocial support although 73.6% (98.5% with perceived need) would accept such an offer. As acceptance of psychosocial support is obviously given, it can be assumed that barriers that continue to exist prevent access to appropriate offices. These barriers may be structural or individual in nature. Furthermore, it should also be emphasized that about half of the patients without a psychosocial distress and/or perceived need are willing to accept an offer of psychosocial support. These results show that preventive psychosocial interventions could meet with a high level of acceptance among cancer patients. Thisquestion should also be examined more closely in future studies.

## Data Availability

The data is available on request from the authors.
